# A cross-sectional observational study of unmet health needs among homeless and vulnerably housed adults in three Canadian cities

**DOI:** 10.1186/1471-2458-13-577

**Published:** 2013-06-13

**Authors:** Niran Argintaru, Catharine Chambers, Evie Gogosis, Susan Farrell, Anita Palepu, Fran Klodawsky, Stephen W Hwang

**Affiliations:** 1 Centre for Research on Inner City Health, Keenan Research Centre in the Li Ka Shing Knowledge Institute, St. Michael’s Hospital, Toronto, ON, Canada; 2 Institute of Mental Health Research, University of Ottawa, Ottawa, ON, Canada; 3 Department of Medicine, University of British Columbia, Vancouver, BC, Canada; 4 School of Geography and Environmental studies, Carlton University, Ottawa, ON, Canada

**Keywords:** Access to care, Homelessness, Housing, Primary care, Public health policy

## Abstract

**Background:**

Homeless persons experience a high burden of health problems; yet, they face significant barriers in accessing health care. Less is known about unmet needs for care among vulnerably housed persons who live in poor-quality or temporary housing and are at high risk of becoming homeless. The objectives of this study were to examine the prevalence of and factors associated with unmet needs for health care in a population-based sample of homeless and vulnerably housed adults in three major cities within a universal health insurance system.

**Methods:**

Participants were recruited at shelters, meal programs, community health centers, drop-in centers, rooming houses, and single room occupancy hotels in Vancouver, Toronto, and Ottawa, Canada, throughout 2009. Baseline interviews elicited demographic characteristics, health status, and barriers to health care. Logistic regression was used to identify factors associated with self-reported unmet needs for health care in the past 12 months.

**Results:**

Of the 1,181 participants included in the analysis, 445 (37%) reported unmet needs. In adjusted analyses, factors associated with a greater odds of reporting unmet needs were having employment in the past 12 months (AOR = 1.40, 95% CI = 1.03–1.91) and having ≥3 chronic health conditions (AOR = 2.17, 95% CI = 1.24–3.79). Having higher health-related quality of life (AOR = 0.21, 95% CI = 0.09–0.53), improved mental (AOR = 0.97, 95% CI = 0.96–0.98) or physical health (AOR = 0.98, 95% CI = 0.96–0.99), and having a primary care provider (AOR = 0.63, 95% CI = 0.46–0.85) decreased the odds of reporting unmet needs.

**Conclusions:**

Homeless and vulnerably housed adults have a similar likelihood of experiencing unmet health care needs. Strategies to improve access to primary care and reduce barriers to accessing care in these populations are needed.

## Background

Approximately 150,000 Canadians and up to 3.5 million Americans experience homelessness in a year [1,2]. For many, homelessness is a dynamic state characterized by shifts between unstable housing and homelessness. Homeless individuals who obtain housing often have difficulty maintaining it due to factors such as financial hardship, physical or mental health problems, substance use, and lack of social support [3,4]. And so, as many as 400,000 individuals in Canada are classified as “vulnerably housed,” referring to persons living in poor-quality, temporary or precarious housing such as single room occupancy (SRO) hotels or rooming houses [5]. Homeless and vulnerably housed individuals suffer from numerous threats to their health [4], high rates of substance abuse and mental illness, and increased mortality compared to low-income individuals in the general population [6-8].

Housing has a substantial impact on mental and physical health; hence, it is an important social determinant of health [9,10]. Higher rates of substance abuse, serious medical issues, and trauma exist among unsheltered homeless adults compared to the general population [11]. Good quality housing would be expected to provide social and personal stability, protection from the elements, privacy and sanitation, and more reliable contact with health care providers, factors which should contribute to improved health and access to care [11]. However, homeless and vulnerably housed individuals may fail to experience these benefits.

Homeless individuals are known to experience poor access to health care [12-15], and, as a result, may experience deterioration in health status, prolonged homelessness, and increased mortality [16]. Barriers to accessing services include financial difficulties, lack of knowledge about where to obtain care, lack of transportation, lack of child care, long waiting times, perceived discrimination in health care settings, and competing priorities for subsistence needs [13,17]. In a 1996 U.S. national survey of nearly 3,000 homeless adults, 24.6% reported being unable to access the health care they needed, and not having health insurance was noted as a significant barrier [13]. A 2006 study of homeless and vulnerably housed individuals noted over 40% of participants visited the emergency department at least once a year, compared with under 30% of people living under the poverty line and approximately 20% of the United States general population [17,18]. Frequent emergency department visits were associated with unstable housing and were partly attributed to food and housing insecurity [17]. However, because up to half of homeless adults in the United States do not have health insurance [13], these findings may not be applicable to countries such as Canada which have publicly funded systems of universal health insurance and significant differences in social services, housing availability, economy and climate.

This study examines factors associated with unmet health needs among homeless and vulnerably housed individuals across three major Canadian cities and whether being vulnerably housed is associated with a lower likelihood of unmet needs for health care compared to being homeless.

## Methods

This study used baseline data from the Health and Housing in Transition (HHiT) study, an ongoing longitudinal study examining housing and health care utilization of homeless and vulnerably housed individuals over a four year period.

### Study setting

Individuals were recruited from Toronto, Ontario (population 2.62 million); Ottawa, Ontario (population 883,391); and Vancouver, British Columbia (population 603,502) [19]. On any given night there are approximately 2,600 homeless individuals in Vancouver, over 900 in Ottawa, and as many as 4,400 in Toronto [20-22]. Low-cost housing alternatives consist largely of licensed and unlicensed rooming houses in Toronto and Ottawa and SRO hotels in Vancouver.

In Canada, publicly funded universal health insurance is provided for all citizens, landed immigrants, and refugees who meet residency requirements. Under these provincially administered health insurance plans, individuals have coverage for all medically necessary health services without patient co-payments [23,24]. These include inpatient hospital services, emergency department visits, outpatient services and some homecare.

### Participants

A total of 1,191 single adults (aged 18 or older who do not live with a partner or dependent child) were recruited throughout 2009. Participants were recruited as part of two discrete samples: homeless adults and vulnerably housed adults. Homelessness was defined as living in a shelter, public space, vehicle, abandoned building, or someone else’s home, and not living one’s own home within the last seven days. Vulnerably housed status was defined as living in one’s own room, apartment, or place, but having been or having two or more moves over the past 12 months. The two-stage sampling method, where recruitment locations were first sampled and then participants were sampled within the individual locations was adapted from Ardilly and Le Blanc (2001) and was further validated in Marpsat and Razafindratsima (2010) [25,26]. Detailed sampling and recruitment methods used for this study have been published previously and are briefly described here [6].

Homeless individuals were recruited at shelters and meal programs. At meal programs, recruitment of homeless people was limited to those who did not use shelters, with their numbers proportionate to the number of homeless persons estimated to sleep on the street in each city [27]. Shelters were randomly selected proportionate to their number of beds, while meal programs were selected randomly based on location and estimated number of individuals who were served weekly. Vulnerably housed individuals were recruited from SRO hotels in Vancouver and rooming houses in Toronto and Ottawa as well as from meal programs, drop-in centers, and community health centers in all three cities. Although recruitment of some homeless and vulnerably housed participants took place in meal programs, participants were classified into the two groups based on the above definitions. Participants provided written informed consent and received $20 CDN for completing the interview. Ethics approval was obtained from the Research Ethics Boards at St. Michael’s Hospital (Toronto), the University of Ottawa, and the University of British Columbia (Vancouver).

### Survey

Structured in-person interviews lasting 60 to 90 minutes were conducted immediately after recruitment. Information was obtained about demographic characteristics, health status, health conditions, and barriers to accessing health care by self-report. The primary outcome measure for this analysis was unmet need for health care, based on the question “During the past 12 months, was there ever a time when you felt that you needed health care but you didn’t receive it?”

Participants self-identified their ethnic background from categories adapted from the Statistics Canada Ethnic Diversity Survey [28]. Individuals were asked whether they had a regular medical doctor/nurse practitioner in the past 12 months. Individuals were also asked whether they had a provincial health insurance number, which is issued to all persons covered by the universal health insurance system.

Health status was assessed using the Short Form 12-item health survey (SF-12), a health status instrument that provides reliable physical and mental health summary measures [29]. Physical component summary (PCS) and mental component summary (MCS) scores were calculated according to the publisher’s specifications, and standardized to the US general population (mean score = 50; SD = 10), with higher scores representing better overall health [6,29]. Individuals were also asked to identify any chronic health conditions lasting six months or more and were diagnosed by a health professional using items adapted from the Canadian Community Health Survey [30]. In an exploratory analysis, self-reported mental health diagnoses were aggregated into three categories: mood disorders (including bipolar disorder, depression, and manic disorder); anxiety disorders (including generalized anxiety disorders, obsessive compulsive disorder, panic disorder, phobias, and posttraumatic stress disorder); and schizophrenia and other psychotic disorders.

Health-related quality of life was measured using the EuroQol (EQ-5D) instrument that scores quality of life based on five dimensions (mobility, usual activity, self-care, pain/discomfort, and anxiety/depression) and provides a global rating of current health using a visual analog scale (VAS) ranging from 0–100 [31,32]. Weighted composite scores were calculated using weights for the United States general population; scores theoretically range between −0.11 and 1.00 on a scale where 0 represents death and 1 represents perfect health [32].

### Statistical analysis

Of 1,191 participants, 10 (0.8%) responded “don’t know,” refused to answer, or had missing data for having unmet health care needs and were excluded from all analyses. Bivariate comparisons were made between participants who reported having unmet needs for health care and those who did not using Student’s t-test for continuous variables and chi-square tests or Fisher’s Exact test (where appropriate) for categorical variables. Logistic regression was used to identify factors associated with reporting unmet needs.

An analysis for gender-specific interactions with age, city, housing status at recruitment, SF-12 scores, self-reported mental health diagnoses, employment over the past 12 months, and having been a victim of a sexual assault revealed no significant interactions. Hence, stratified analyses by gender were not performed.

The multivariate regression model included all factors that could plausibly contribute to unmet needs for health care. Results were considered significant at the alpha = 0.05 level. A total of 142 (11.9%) participants were not included in the final multivariate model due to missing values for any of the 18 variables in the model. Final analyses collapsed data from all three cities into a single population, as stratified analyses by city did not yield significant differences. Analysis was performed using SPSS 19.0 for Windows (SPSS Inc., Chicago IL).

## Results

In total, 445 participants (37.7%) reported an unmet need for health care in the past 12 months. Table 1 summarizes the characteristics of participants with and without unmet needs for health care. The average lifetime duration of homelessness reported was 5.1 years (SD = 6.0 years), and there were no significant demographic differences between the groups with and without unmet needs based on housing status, age, gender, city, education, or employment status (Table 1). However, significant differences were noted for immigrant status, racial/cultural group, and lifetime duration of homelessness.

**Table 1:** Characteristics of participants with and without self-reported unmet needs for care in Vancouver, Toronto, and Ottawa, Canada, 2009

	**All participants (n = 1181)**	**Participants with unmet needs (n = 445)**	**Participants without unmet needs (n = 736)**	***p***
Demographics, n (%)				0.71
Homeless	592 (50.1)	220 (49.4)	372 (50.5)	
Vulnerably housed	589 (49.9)	225 (50.6)	364 (49.5)	
Age group, n (%)				0.52
<30 years	159 (13.5)	62 (14.0)	97 (13.2)	
30–39 years	291 (24.7)	116 (26.2)	175 (23.8)	
40–49 years	439 (37.2)	166 (37.5)	273 (37.1)	
≥50 years	290 (24.6)	99 (22.3)	191 (26.0)	
Gender, n (%)				0.25
Male	773 (65.7)	286 (64.4)	487 (66.4)	
Female	386 (32.8)	154 (34.7)	232 (31.7)	
Transgender	18 (1.5)	4 (0.9)	14 (1.9)	
City, n (%)				0.16
Vancouver	391 (33.1)	160 (36.0)	231 (31.4)	
Toronto	396 (33.5)	136 (30.6)	260 (35.3)	
Ottawa	394 (33.4)	149 (33.5)	245 (33.3)	
Born in Canada, n (%)	992 (84.5)	386 (87.1)	606 (82.9)	0.05
Racial/cultural group, n (%)				<0.01
White	712 (62.2)	282 (65.0)	430 (60.5)	
Black/African-Canadian	106 (9.3)	33 (7.6)	73 (10.3)	
First Nations/Aboriginal	204 (17.8)	79 (18.2)	125 (17.6)	
Mixed ethnicity	64 (5.6)	30 (6.9)	34 (4.8)	
Other	59 (5.2)	10 (2.3)	49 (6.9)	
Highest level of education, n (%)				0.38
Some post-secondary education or more	375 (32.0)	148 (33.4)	227 (31.1)	
Completed high school or equivalent	274 (23.4)	94 (21.2)	180 (24.7)	
Some high school	523 (44.6)	201 (45.4)	322 (44.2)	
Employed past 12 months, n (%)	468 (39.7)	186 (41.9)	282 (38.4)	0.23
Lifetime duration of homelessness (yrs), mean (SD)	5.1 (6.0)	5.5 (6.3)	4.9 (5.8)	0.07
EQ-VAS score (perceived state of health), mean (SD)	61.5 (21.8)	55.5 (22.2)	65.0 (20.8)	<0.01
EQ-5D index score, mean (SD)	0.7 (0.2)	0.7 (0.2)	0.8 (0.2)	<0.01
Been victim of sexual assault over past 12 months, n (%)	104 (8.9)	55 (12.4)	49 (6.7)	<0.01
Has a provincial health card number, n (%)	991 (86.9)	365 (85.5)	626 (87.7)	0.29
Has unmet needs for mental care, n (%)	275 (23.4)	183 (41.4)	92 (12.6)	<0.01
Has primary care provider, n (%)	713 (60.5)	252 (56.8)	461 (62.7)	0.04
SF-12 PCS, ^1^ mean (SD)	44.5 (11.3)	41.4 (10.9)	46.4 (11.1)	<0.01
SF-12 MCS, mean (SD)	39.1 (13.1)	34.9 (12.2)	41.6 (12.9)	<0.01
Number of chronic health conditions, ^2^ n (%)				<0.01
0	150 (12.7)	30 (6.7)	120 (16.3)	
1	248 (21.0)	69 (15.5)	179 (24.3)	
2	195 (16.5)	65 (14.6)	130 (17.7)	
≥ 3	588 (49.8)	281 (63.1)	307 (41.7)	
Diagnosed with a mental health disorder, n (%)	600 (51.5)	260 (59.2)	340 (46.9)	<0.01

^1^On a scale where 50 is the mean and 10 is the standard deviation in the US general population.

^2^Chronic health conditions include: high blood pressure; heart disease; asthma; COPD (includes emphysema and chronic bronchitis); cirrhosis; Hepatitis B or C; intestinal or stomach ulcers; urinary incontinence; bowel disorders; arthritis; problems walking, lost limb, or other physical handicap; HIV/AIDS; epilepsy; fetal alcohol syndrome or fetal alcohol spectrum disorder; head injury; glaucoma; cataracts; cancer, diabetes; or anemia.

Participants who reported unmet needs for health care also had a significantly lower mean EQ-VAS scores (55.5 vs. 65.0, *p* < 0.01) and mean EQ-5D index score (0.7 vs. 0.8, *p* < 0.01) compared to those without unmet needs. They were significantly more likely to report having three or more chronic health conditions than those without unmet needs (63.1% vs. 41.7%, *p* < 0.01). Additionally, a history of a previous mental health diagnosis was significantly more common among participants with unmet needs for health care (59.2% vs. 46.9%, *p* < 0.01). Not surprisingly, participants with unmet needs for care were more likely to report also having an unmet need for mental health care (41.4% vs. 12.6%, *p* < 0.01).

Of the 736 participants without unmet needs for health care, 92 (12.5%) reported having unmet needs for mental health care in the same time period while 183 (66.5%) of those who indicated having unmet needs for mental health care also reported unmet needs for care. Overall, 23.4% of participants reported unmet mental health care needs.

Table 2 summarizes the results of bivariate and multivariate logistic regression models identifying factors associated with reporting unmet needs. Seven characteristics were significantly associated with increased likelihood of reporting unmet needs for health care in unadjusted regression models: being a victim of sexual assault in the past 12 months, having been diagnosed with a mental health disorder, having lower EQ-5D index or VAS scores, having lower SF-12 PCS and MCS scores, and having more than one chronic health condition.

**Table 2:** Factors associated with unmet needs for health care among homeless and vulnerably housed participants in Toronto, Ottawa and Vancouver, Canada, 2009

	**Bivariate models**		**Multivariate model**	
	**OR (95% CI)**	***p***	**AOR (95% CI)**	***p***
Demographics, n (%)				
Homeless (Ref)	1.00		1.00	
Vulnerably housed	1.05 (0.83, 1.32)	0.71	1.09 (0.82, 1.45)	0.56
Age group, n (%)				
<30 years (Ref)	1.00		1.00	
30-39 years	1.04 (0.70, 1.54)	0.86	0.87 (0.54, 1.40)	0.56
40–49 years	0.95 (0.66, 1.38)	0.79	0.74 (0.46, 1.18)	0.20
≥50 years	0.81 (0.54, 1.21)	0.31	0.63 (0.37, 1.05)	0.07
Gender, n (%)				
Male (Ref)	1.00		1.00	
Female	1.13 (0.88, 1.45)	0.34	0.84 (0.60, 1.19)	0.32
Transgender	0.49 (0.16, 1.49)	0.21	0.24 (0.04, 1.05)	0.06
City, n (%)				
Vancouver (Ref)	1.00		1.00	
Toronto	0.76 (0.57, 1.01)	0.06	1.10 (0.77, 1.59)	0.60
Ottawa	0.88 (0.66, 1.17)	0.37	0.90 (0.63, 1.27)	0.55
Born in Canada, n (%)	1.40 (1.00, 1.96)	0.05	0.89 (0.53, 1.49)	0.65
Racial/cultural group, n (%)				
White (Ref)	1.00		1.00	
Black/African-Canadian	0.69 (0.45, 1.07)	0.10	0.76 (0.41, 1.39)	0.37
First Nations/Aboriginal	0.96 (0.70, 1.33)	0.82	0.80 (0.54, 1.17)	0.24
Mixed ethnicity	1.35 (0.81, 2.25)	0.26	1.34 (0.68, 2.25)	0.49
Other	0.31 (0.15, 0.62)	<0.01	0.28 (0.11, 0.69)	<0.01
Highest level of education, n (%)				
Some post-secondary education or higher (Ref)	1.00		1.00	
Completed high school or equivalent	0.80 (0.58, 1.11)	0.18	0.67 (0.46, 1.00)	0.05
Some high school	0.96 (0.73, 1.26)	0.75	0.93 (0.67, 1.29)	0.66
Employed in past 12 months, n (%)	1.16 (0.91, 1.47)	0.23	1.40 (1.03, 1.91)	0.03
Lifetime duration of homelessness (years), mean (SD)	1.02 (0.99, 1.04)	0.07	1.01 (0.98, 1.03)	0.59
VAS score (perceived state of health), mean (SD)	0.98 (0.97, 0.99)	<0.01	1.00 (0.99, 1.01)	0.51
EQ_5D, mean (SD)	0.06 (0.03, 0.11)	<0.01	0.21 (0.09, 0.53)	<0.01
Been victim of a sexual assault over past 12 months, n (%)	1.95 (1.30, 2.93)	<0.01	1.23 (0.73, 2.08)	0.44
Has a provincial health card number, n (%)	0.83 (0.58, 1.17)	0.29	0.68 (0.44, 1.04)	0.08
Has a primary care provider, n (%)	0.78 (0.61, 0.99)	0.04	0.63 (0.46, 0.85)	<0.01
SF-12 PCS, mean (SD)	0.96 (0.95, 0.97)	<0.01	0.98 (0.96, 0.99)	<0.01
SF-12 MCS, mean (SD)	0.96 (0.95, 0.97)	<0.01	0.97 (0.96, 0.98)	<0.01
Number of chronic health conditions, n (%)				
0 (Ref)	1.00		1.00	
1	1.54 (0.95, 2.51)	0.08	1.26 (0.71, 2.22)	0.43
2	2.00 (1.22, 3.29)	<0.01	1.50 (0.83, 2.69)	0.18
≥ 3	3.66 (2.38, 5.64)	<0.01	2.17 (1.24, 3.79)	<0.01
Ever diagnosed with a mental health disorder, n (%)	1.65 (1.29, 2.09)	<0.01	1.13 (0.84, 1.53)	0.41

In the multivariate logistic regression model, housing status did not significantly alter the likelihood of reporting unmet needs for health care. Factors that increased the likelihood of unmet needs for health care in adjusted models included being employed in the past 12 months (AOR = 1.41, 95% CI = 1.03, 1.91) and having three or more chronic health conditions (AOR = 2.17, 95% CI = 1.24, 3.79). Factors that decreased the likelihood of unmet needs for health care include having a higher EQ-5D score (AOR = 0.21, 95% CI = 0.09, 0.53), having a higher SF-12 PCS score (AOR = 0.98, 95% CI = 0.96, 0.99) or MCS score (AOR = 0.97, 95% CI = 0.96, 0.98), completing high school or equivalent (AOR = 0.67, 95% CI = 0.46, 1.00) and having a primary health care provider (AOR = 0.63, 95% CI = 0.46, 0.85).

An exploratory bivariate analysis of participants reporting one or more previous mental health diagnoses is summarized in Table 3 and identifies significantly increased likelihood of reporting unmet need for health care in participants with mood disorders (OR = 1.73, 95% CI = 1.36, 2.20) and anxiety disorders (OR = 1.96, 95% CI = 1.48, 2.57). Schizophrenia or other psychotic disorders were not associated with increased likelihood of reporting unmet needs (OR = 0.82, 95% CI = 0.50, 1.33).

**Table 3:** Unmet needs for health care in participants diagnosed with mental health conditions

**Mental health disorder**	**All participants (n = 600)**^**3**^	**Unmet needs for care (n = 260)**	**No unmet needs for care (n = 340)**	**Bivariate Model OR (95% CI)**	***p***
Mood disorder (including bipolar disorder, depression, manic disorder)	453 (38.6%)	207 (46.5%)	246 (33.5%)	1.73 (1.36, 2.20)	<0.01
Anxiety disorder (including generalized anxiety disorder, OCD, panic disorder, phobia, PTSD)	256 (21.7%)	129 (29.0%)	127 (17.3%)	1.96 (1.48, 2.57)	<0.01
Schizophrenia and other psychotic disorders	78 (6.6%)	26 (5.8%)	52 (7.1%)	0.82 (0.50, 1.33)	0.41

^3^Participants were allowed to report more than one mental health diagnosis, therefore total does not equal sum of column.

## Discussion

In this study of homeless and vulnerably housed adults in three Canadian cities, over one-third (37%) of participants reported unmet needs for health care in the past 12 months. This is a higher proportion than a similar Toronto study of homeless people that noted a prevalence of unmet needs for health care of 22% among single adult women and 14% among single adult males [33]. This discrepancy may be due to the previous study focusing specifically on care from a doctor or nurse in the past 12 months, while the current study investigated perceived health care needs more generally. The prevalence of unmet needs in our sample is, however, similar to other studies that have investigated unmet needs among homeless populations in the United States [13,15,34].

An important finding of our analysis was that being vulnerably housed was not associated with a lower likelihood of having unmet needs for health care when compared to being homeless. This finding is supported by prior research on housing and health status, which suggests that homeless and vulnerably housed individuals are intersecting and dynamic populations with equally poor health status and similar barriers to accessing care [34]. This study suggests that provision of housing is not sufficient in and of itself to ensure health care access. Our findings highlight the importance of access to stable, secure, not overcrowded, affordable housing with appropriate supports to both homeless and vulnerably housed populations.

Having worse mental or physical health and having a greater number of chronic health conditions were significantly associated with an increased likelihood of having unmet needs for health care. This association is not surprising, since a greater need for health care would be expected to increase the risk of having unmet needs for care, especially given the numerous barriers to accessing health care that homeless and vulnerably housed people experience [34,35].

Our exploratory findings suggest that the ability or perceived ability to access care effectively may be particularly problematic for individuals with certain mental health conditions, such as mood and anxiety disorders. Alternatively, individuals with schizophrenia or other psychotic disorders may have a tendency to underestimate their need for care and thus report fewer unmet needs. Further research will be required to better delineate these differences.

The majority (86.9%) of participants in our study reported having a provincial health number, either in their possession or on record at a health care site (e.g., doctor’s office, hospital). Having a provincial health insurance number is essential to accessing most medically necessary services in Canada. Although there was a tendency for participants without a provincial health number to report unmet needs for care, the trend was not statistically significant. It is also possible that participants may be accessing health care through low-barrier sites such as shelter-based clinics or community health centers, which may not require clients to present proof of health insurance.

In the adjusted analyses, as with the bivariate models, being vulnerably housed as opposed to homeless at recruitment was not associated with having unmet needs for care; therefore, the two samples were combined into a single analytic group. Having a primary care provider significantly reduced the likelihood of having unmet needs for care, even when adjusting for potential confounders and covariates, highlighting the role of a primary care provider as a gateway to accessing healthcare services. There was no independent association between being a victim of sexual assault and unmet needs for health care, which differs from a previously identified association between unmet health care needs and sexual victimization; however, gender differences were not explored in either analysis [33]. Interestingly, participants who were employed in the previous 12 months were more likely to have unmet needs for health care, possibly due to competing priorities between health and employment, incompatibility between the hours of employment and hours of care, difficulty keeping appointments, or decreased exposure to community and shelter-based health care programs [35].

Participants’ health status remained a key factor in the likelihood of reporting unmet needs for health care, particularly when identified by the SF-12 mental and physical summary measures and self-reported chronic health conditions. A lower health-related quality of life, identified by the EQ-5D instrument, was also significantly associated with a higher likelihood of reporting unmet needs for care.

Taken together, our findings suggest that, despite the universal provision of health insurance, barriers still exist to accessing health services, particularly among those individuals who have extremely poor health status and are most in need of health care. These barriers may be related to non-financial factors such as lack of knowledge about where to obtain care, lack of transportation, lack of child care, long waiting times, perceived discrimination in health care settings, and competing priorities for subsistence needs [13,15,36,37]. Addressing potential barriers to accessing health care in Canada will also require the creation of policies that acknowledge the social determinants of health, in particular the provision of stable housing [36].

### Limitations

Health status and health care access were assessed on the basis of self-report. Although self-reported needs for care are a recognized indicator of health care access, they present a subjective participant viewpoint. Previous research has shown homeless populations tend to underreport health issues [38]. Additionally, when reporting unmet needs for health care, individuals may not be aware of what care that is in fact available to them or may expect therapy that is not necessarily appropriate. Recruitment of some vulnerably housed participants from sites that provide health services (e.g., community health centres) may have resulted in a slight over-representation in our sample of vulnerably housed individuals who are better able to access care.

The criteria used to enrol study participants resulted in significant similarities between the groups of homeless and vulnerably housed participants. However, this result highlights the dynamic nature of homelessness and the considerable overlap between individuals who reside in low quality housing, in shelters or on the streets [6]. Geographic factors and differences in health care provision across cities were not specifically addressed in this study. Gender-specific analyses, which may shed light on the unique health care needs of women versus men, were not performed. Self-reported chronic health conditions were required to be diagnosed by a health professional, which may result in a under-reporting of chronic health conditions in participants who were unable to access care. Further studies and analysis is required to effectively assess the role of mental health and specific mental health disorders in unmet needs for health care.

Lastly, the study’s cross-sectional design is unable to explore the casual association between changes in housing status and quality of housing on an individual’s ability to access care. Further research by our group will investigate changes in health status as well as health care access and associations with housing status using longitudinal data from the HHiT study.

## Conclusions

This study identified no differences in the likelihood of unmet health care needs between homeless and vulnerably housed adults, highlighting that both populations face significant challenges in accessing health care. Homeless and vulnerably housed individuals with multiple chronic health conditions, worse health status, or no primary care provider were more likely to report unmet needs for health care. It is therefore important to develop policies and programs that are accessible, available, and appropriate for homeless and vulnerably housed individuals in order to meet their health care needs. Despite Canada’s universal health insurance system, homeless and vulnerably housed populations face barriers to meeting health care needs. Future studies should identify the types of health care that are lacking and effective strategies to reduce barriers to accessing care.

## Competing interests

The authors declare that they have no competing interests.

## Authors’ contributions

All of the authors contributed to the study concept and design. SH, SF, AP, FK and EG acquired the data. NA and CC performed the statistical analysis. SF, AP, FK and EG contributed to the analysis. They, along with SH and CC and interpreted the data. NA drafted the manuscript, and all of the authors critically revised it for important intellectual content and approved the final version submitted for publication.

## Pre-publication history

The pre-publication history for this paper can be accessed here:

http://www.biomedcentral.com/1471-2458/13/577/prepub
